# FEEDSETUP: feeding difficulties spectrum evaluation and treatment up-to-date proposal

**DOI:** 10.1016/j.jped.2026.101610

**Published:** 2026-09-18

**Authors:** Mauro Fisberg, Carlos A. Nogueira-de-Almeida, Tulio Konstantyner

**Affiliations:** a Universidade Federal de São Paulo (UNIFESP), Escola Paulista de Medicina, Departamento de Pediatria, São Paulo, SP, Brazil; b Cenda Instituto Pensi, São Paulo, SP, Brazil; c Universidade Federal de São Carlos (UFSCar), Departamento de Medicina, São Carlos, SP, Brazil; d Universidade Federal de São Paulo (UNIFESP), Departamento de Pediatria, São Paulo, SP, Brazil

**Keywords:** Feeding behavior, Feeding and eating disorders of childhood, Food fussiness, Avoidant restrictive food intake disorder, Autism spectrum disorder, Diagnosis

## Abstract

**Objective:**

To organize current knowledge and propose a comprehensive, treatment-focused classification system, called FEDSETUP, that guides clinicians through a stepwise, multidisciplinary approach to pediatric feeding difficulties, emphasizing intervention intensity matched to clinical need and severity.

**Sources:**

Development of the FEEDSETUP framework was grounded in a systematic approach to evidence synthesis, following PRISMA principles adapted for narrative framework development. Given the multidimensional nature of pediatric feeding difficulties—spanning medical, nutritional, behavioral, and developmental domains—a structured PICO framework was employed to guide literature identification and selection.

**Summary of the findings:**

FEEDSETUP integrates a systematic initial assessment encompassing medical history, dietary evaluation, physical examination, and anthropometry. It categorizes feeding difficulties into distinct yet overlapping domains, including underlying organic disease, typical neophobia, micronutrient deficiency, difficulty concentrating on food, food phobia, and food selectivity. The framework prioritizes primary healthcare as the first line of intervention, with clear criteria for escalation to specialized multidisciplinary teams for more complex or severe cases, including those with Autism Spectrum Disorder comorbidity or Avoidant/Restrictive Food Intake Disorder.

**Conclusions:**

FEEDSETUP offers a comprehensive, integrated, and treatment-oriented approach to pediatric feeding difficulties, moving beyond diagnosis-centric models to provide a structured pathway for effective and individualized care across the spectrum of severity. The algorithm provides a practical, evidence-based roadmap for clinicians to ensure timely identification of associated behavioral, nutritional, and organic abnormalities. It facilitates interdisciplinary assessment, reduces diagnostic delays, and promotes coordinated care by matching intervention intensity to clinical presentation, thereby avoiding both under-treatment and over-medicalization.

## Introduction

Feeding difficulties (FD) affect 25 to 45% of typical children and up to 80% of children with developmental problems [1,2]. A recent Brazilian study that interviewed 1000 mothers from all over the country showed that 62% of them reported feeding difficulties [3]. Despite the high prevalence, there is significant confusion regarding terminology, diagnostic criteria, and appropriate treatment pathways. A child who "does not eat" can receive different labels depending on the specialist who evaluates him, whether it is a psychiatrist (Avoidant/Restrictive Food Intake Disorder - ARFID), pediatric gastroenterologist (eating disorder), behavioral psychologist (food selectivity), or developmental specialist (food neophobia) [4,5]. This fragmentation creates a number of clinical problems, leading to delayed or inadequate referrals, as primary care providers have difficulty determining which specialty is the most appropriate. At the same time, families often receive conflicting information from different providers who use different diagnostic frameworks. The lack of coordinated multidisciplinary care results in fragmented treatment approaches. Finally, there is difficulty in accessing appropriate interventions when the diagnostic terminology does not correspond to the available service structures [6]. The situation is further complicated by the overlap with Autism Spectrum Disorder (ASD), where eating problems are common [7], but which can be caused by different underlying mechanisms that require different treatment approaches [8,9].

Prior to 2013, feeding problems in children without developmental failure were poorly characterized in psychiatric nosology. The DSM-IV only included "Childhood Eating Disorder or Early Childhood," which required onset before age 6 and associated weight loss [10]. Many children with significant feeding restrictions but adequate growth had no formal diagnosis. The pediatric literature described "selective feeding", "refusal to feed" and "selective feeding" without standardized criteria [11,12]. This created a diagnostic gap where children with clinically significant feeding problems that did not result in growth failure remained unrecognized by formal grading systems. In parallel with psychiatric advances, pediatric specialists have developed the Pediatric Eating Disorder (PED) framework, emphasizing multi-domain dysfunction, considering medical, nutritional, dietary skills, and psychosocial aspects rather than focusing solely on nutritional or growth outcomes [1,13]. This model facilitates interdisciplinary assessment and treatment planning by providing a common language across specialties.

Seeking to systematize the accumulated knowledge on the subject, Kerzner et al. [2,14] proposed a classification focused especially on diagnosis and warning signs (red flags). According to these authors, most cases would fall into one of the following situations: misinterpretation by parents, agitated child with little appetite, emotionally compromised or neglected child, presence of organic disease, highly selective ingestion, food phobia, and crying that interferes with eating. One of the great advances of this system was the valorization of several aspects that, although very different, had as an outcome the difficulty of eating. Organic or psychic diseases that interfere with eating began to be considered.

Other classification and consensus proposals have been published in more recent years [1,[15], [16], [17], [18]]. An important gap in all previous attempts at classification lies in the fact that they do not lead to treatment. Considering that very different conditions can have eating difficulties as an outcome, therapeutic strategies must be linked to the diagnosis.

### Rationale for the development of FEEDSETUP

**Figure 1:**
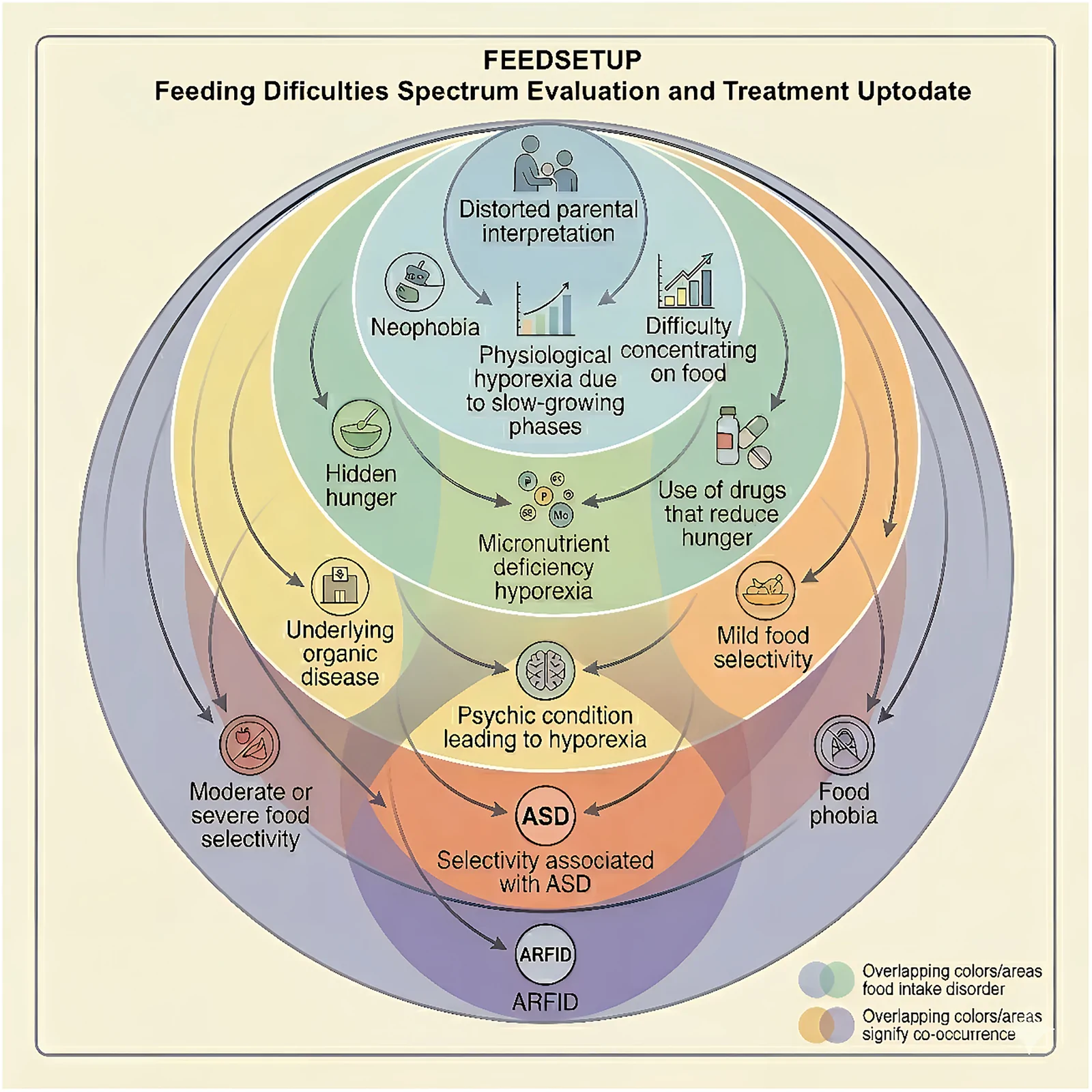
The FEEDSETUP framework.

**Figure 2:**
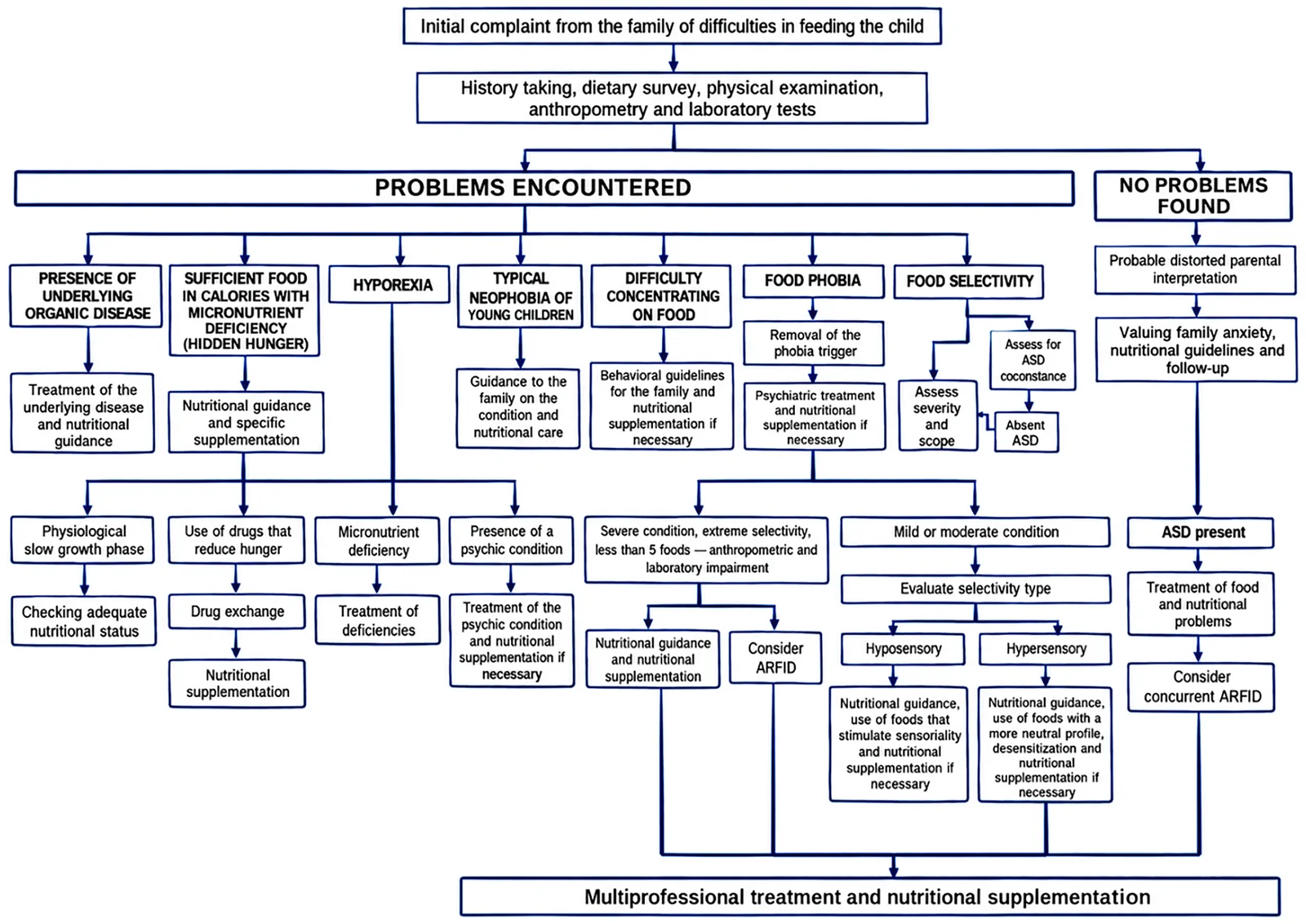
The FEEDSETUP treatment algorithm. ASD, Autism Spectrum Disorder; ARFID, Avoidant/Restrictive Food Intake Disorder.

The objective of the FEEDSETUP approach is to organize current knowledge to present a broader classificatory approach, which is understood as a spectrum associated with different severities, but with the possibility of relevant intersections between diagnoses. The authors named it FEEDSETUP (Feeding Difficulties Spectrum Evaluation and Treatment UpToDate Proposal), and it is shown in Figure 1. FEEDSETUP integrates a systematic initial assessment encompassing medical history, dietary evaluation, physical examination, and anthropometry. It categorizes feeding difficulties into distinct yet overlapping domains, including underlying organic disease, typical neophobia, micronutrient deficiency, difficulty concentrating on food, food phobia, and food selectivity (hypersensorial and hyposensorial subtypes). The framework prioritizes primary healthcare as the first line of intervention, with clear criteria for escalation to specialized multidisciplinary teams for more complex or severe cases, including those with Autism Spectrum Disorder (ASD) comorbidity or Avoidant/Restrictive Food Intake Disorder (ARFID). Finally, the classification proposal seeks to lead to an algorithm in which the outcome is the specific treatment (Figure 2).

### Evidence base and selection of literature

The development of the FEEDSETUP framework was grounded in a systematic approach to evidence synthesis, following PRISMA (Preferred Reporting Items for Systematic Reviews and Meta-Analyses) principles adapted for narrative framework development. Given the multidimensional nature of pediatric feeding difficulties — spanning medical, nutritional, behavioral, and developmental domains — the authors employed a structured PICO (Population, Intervention, Comparison, Outcome) framework to guide literature identification and selection. The databases consulted were PubMed/MEDLINE, Scopus and the Cochrane Library, including articles in English, Portuguese and Spanish using the descriptors: Feeding Behavior; Feeding and Eating Disorders of Childhood; Food Fussiness; Avoidant Restrictive Food Intake Disorder; Autism Spectrum Disorder. The authors also screened the reference lists of retrieved articles. Publications from the last 50 years were initially considered, giving priority to systematic reviews, meta-analyses, clinical practice guidelines and consensus statements. The authors excluded papers regarding eating disorders driven by weight and shape concerns (anorexia nervosa, bulimia nervosa). The literature review was registered in the PROPSERO System (CRD420261373752). Table 1 lists, for each of the seven domains of the framework, the key supporting references with study design, population studied, and level of evidence, together with an explicit indication of the domains for which the recommendations rest on expert consensus rather than on controlled studies. No formal risk-of-bias assessment was performed; the table reports the highest level of evidence identified for each domain and makes explicit the domains resting on expert opinion.

**Table 1:** Evidence supporting each FEEDSETUP domain.

Domain	Key references	Best available evidence
Framework and terminology	1, 17	Expert consensus statements
Absence of true feeding problem	28–50	Systematic review [49]; cohort and observational studies
Underlying organic disease	26, 51	Narrative reviews; established clinical practice
Typical neophobia	27, 52, 73	Systematic review of qualitative studies [73]
Micronutrient deficiency	53	Prevalence and observational studies
Difficulty concentrating on food	27, 45, 54–57	Observational studies; expert opinion
Food phobia	52, 58	Case series; extrapolation from anxiety-disorder management
Hyporexia	59–72	Observational and pharmacological reviews; no controlled trials
Food selectivity, general	9, 73–105	Meta-analysis [9]; systematic review of oral-motor assessment [79]
Hypersensorial selectivity	106–115	Systematic review [111]; randomized trial [114]
Hyposensorial selectivity	116–139	Systematic review and meta-analysis [130]; systematic review [132]; randomized pilot trial [5]

### What Feedsetup adds

Existing frameworks classify feeding difficulties for diagnostic purposes and stop there. The Kerzner classification organizes presentations by parental feeding style and child behavior; the Pediatric Feeding Disorder consensus defines dysfunction across medical, nutritional, feeding-skill and psychosocial domains; [1] the DSM-5 criteria for ARFID establish a categorical psychiatric threshold. All three answer the question “what does this child have?”. FEEDSETUP answers a different question: “what should be done, by whom, and at what level of care?”. It treats feeding difficulties as a continuum rather than a set of discrete entities, recognizes overlap between categories as the rule rather than the exception, includes parental misperception without child pathology as a formal category, assigns an explicit and continuing role to primary care, and attaches to each category an indicated intervention and an operational threshold for escalation. Its endpoint is therefore a management decision rather than a label. Table 2 shows the terminology adopted in FEEDSETUP.

**Table 2:** Standardized terminology adopted in FEEDSETUP.

Term	Definition adopted	Distinguishing feature
Food neophobia	Reluctance to accept unfamiliar foods; physiological between ∼2 and 6 years	Refusal restricted to novelty; familiar foods accepted
Food selectivity	Persistent restriction of repertoire by type, texture, color or presentation	Refusal extends to previously accepted foods; repertoire narrows
— hypersensorial subtype	Selectivity driven by excessive sensory responsiveness	Aversion to strong flavors, odours, mixed textures
— hyposensorial subtype	Selectivity driven by reduced sensory responsiveness	Preference for intense flavors; weak hunger signaling
Hyporexia	Reduced appetite and volume, without restriction of food types	Eats little, but eats variedly
Food phobia	Persistent fear of a food or of eating, usually post-aversive event	Anxiety disproportionate to stimulus; identifiable trigger
Restrictive eating behavior	Descriptive umbrella term for reduced intake or repertoire	Descriptive only; not a diagnosis
ARFID	DSM-5 disorder with nutritional, weight or psychosocial impact, without body-image disturbance	Meets categorical threshold; requires specialized care
Pediatric feeding disorder	Age-inappropriate oral intake with medical, nutritional, skill or psychosocial dysfunction [1]	Multi-domain functional definition

### Initial approach

The initial assessment of feeding difficulties in children is a fundamental part of pediatric practice. It enables the early identification of associated behavioral, nutritional, and organic abnormalities. This approach should be systematic and comprehensive, incorporating detailed medical history, dietary assessment, thorough physical examination and anthropometric measurements, as well as targeted laboratory investigations where indicated [1,19].

The medical history should cover gestational and perinatal factors, the history of breastfeeding and the introduction of solid foods, and neuropsychomotor development. The main complaint must be characterized, including the duration and pattern of food refusal, selectivity, sensory aversions, behaviors during meals and possible signs of dysphagia or pain. Psychosocial factors, family dynamics, parenting practices and the feeding environment should also be investigated, as these elements frequently influence children’s eating behavior [[20], [21], [22]].

A dietary assessment should be conducted in a structured manner using either a 24-hour dietary recall, a food diary, or food frequency questionnaires. This stage enables the quantitative and qualitative adequacy of the diet to be evaluated, as well as dietary diversity and the intake of macronutrients and micronutrients. It also allows inappropriate patterns to be identified, such as excessive consumption of ultra-processed foods or dietary monotony. The analysis should consider the age group and current nutritional recommendations [21,23].

A comprehensive physical examination should be performed, paying particular attention to signs of malnutrition or being overweight, and to skin and mucosal changes that could indicate nutritional deficiencies, such as pallor, angular cheilitis and glossitis. Oral abnormalities should also be assessed, including dental caries, malocclusion and ankyloglossia. An assessment of general health and development should also be performed. Where possible, direct observation of the child during feeding can provide valuable information on motor and behavioral aspects [21].

Anthropometric assessment is essential and should include weight and height/length measurements, as well as calculation of body mass index (BMI) for age. Where necessary, it should also include circumference and skinfold thickness measurements. These data should be plotted on the relevant growth charts to enable identification of deviations such as underweight, short stature or overweight, and analysis of growth velocity [23,24].

Requests for laboratory tests should be tailored to the individual patient, based on the clinician's findings. In selected cases, tests such as a complete blood count, ferritin, serum iron, vitamin B12, folic acid, zinc, a biochemical profile, thyroid function tests and inflammatory marker tests may be indicated. Further investigation may be necessary if underlying organic diseases are suspected, such as food allergies, coeliac disease or gastrointestinal disorders [1,2].

In summary, an initial assessment of feeding difficulties in pediatrics should be comprehensive and child-centered. It should consider the child’s family context and integrate clinical, nutritional and behavioral data. This approach facilitates the formulation of sound diagnostic hypotheses and enables the planning of timely and effective interventions [1,2,25].

### Treatment-focused classification

Feeding difficulties in children and adolescents constitute a heterogeneous spectrum of conditions with organic, behavioral, and sensory etiologies that often overlap. These conditions range from distorted parental perceptions to hyporexia and extend to severe cases of selective eating and/or ARFID, including ASD [2,26,27].

A clinical approach based on the etiology of feeding difficulties allows for objective and efficient organization of assessments and targeting of interventions, reducing diagnostic delays and inappropriate interventions [2]. In this context, the authors developed FEEDSETUP, which classifies feeding difficulties into main categories with a direct focus on clinical decision-making, as described below.

#### Absence of problems encountered

After the initial full assessment has been carried out, there are often no problems with the feeding process. Dietary surveys show adequate intake, physical examination without alterations, anthropometry within normal parameters, and laboratory tests without alterations. In these cases, it is evident that, from the child's point of view, no problems were found that require treatment. However, it is very important to understand that, even in these cases, there is effectively a "feeding difficulty". The parents' perception that the child is not eating correctly is already a real difficulty. This process often generates intense family anxiety, which can often lead to caregivers becoming ill [2]. At the same time, the perception, even if mistaken, that the child is not eating properly can lead to intense pressure exerted on the child, often accompanied by verbal and even physical aggression [28].

Some studies have already shown that there are perceptible psychosocial repercussions on the child's future life and that they are consequences of the imbalance between the parents' expectations and the children's eating behavior. Mascola et al. [29] evaluated psychosocial characteristics of picky eater children and found the presence of fights between parents and children during the meal; discussions about food preferences; a tendency to limit the consumption of certain foods but, surprisingly, this trend manifested itself in relation to "non-sweet" foods; high frequency with which the subject "my child's feeding" is addressed by the family in social conversations; and tendency in the preparation of food separately for the child. Chattoor et al. [30] studying picky eater children, they found that, when evaluated through cognitive development tests, when compared to children without feeding difficulties, their performance in terms of mental development score was significantly lower. In two other studies from the same group, the authors found higher scores for several negative aspects, such as more difficult and irregular temperament, negativity, dependence and agitation, and insecurity [31,32] Galloway et al. [33] found signs of anxiety in selective girls with phobia. In another study by the same group [34], the authors found that picky eater children tend to suffer intense pressure from their mothers to eat and found that this pressure was even greater when the mother's own consumption of fruits and vegetables was low. They were also able to observe that, once pressured to eat, consumption was even lower. This pressure extended to pediatricians and health professionals, who tended to reinforce this dimension of pediatric care, aggravating the process of difficulty in feeding. Other authors [[35], [36], [37], [38]] have also studied this aspect and reached similar conclusions, demonstrating that the parental model is more relevant and more efficient than pressure and coercion to increase food consumption quantitatively and qualitatively. Van der Horst [39] suggests that the issue of pleasure should always be linked to the child's feeding process, and excessively controlling practices on the part of parents can harm the family environment, generating negative feelings about food, reducing the pleasure of eating, and aggravating eating difficulties. Micali et al. [40] found that selectivity was the condition most strongly linked to negative behavioral and psychological aspects, such as behavioral and emotional disorders, as well as somatization processes. Jacobi [41] points out that, among these aspects, issues related to internalization, such as difficulties in apprehending healthy behaviors demonstrated by the family and externalization, inappropriately expressing their feelings and desires, seem more evident as behavioral disorders for this group. Jacobi et al. [42] found that children with selectivity had less affective attachment with their parents. Schimid et al. [43] followed 4427 children from birth to 56 months and found that those who had difficulty feeding at five months were more likely to have deficits in adaptive behaviors and social skills at preschool age. Carruthe et al. [44] found that the use of persuasion attempts was common among mothers of picky eater children when compared to controls; They also found that they tended to label their children as "troubled" and prepare them different food. Still in relation to parents, Sanders et al. [28] comparing 19 preschool children with feeding difficulties and 29 healthy ones, found that the parents of the former were more negative and coercive in the feeding process, consistently making aversive comments during meals. Several studies have evaluated the association between parental behavior and infant food selectivity and have observed that controlling parental strategies (coercion, threats, etc.) create a negative atmosphere around eating, preventing the development of pleasure with eating and, consequently, provoking or reinforcing selective behavior [[45], [46], [47], [48], [49], [50]] Van der Horst [39] conducted a study to assess whether improving the pleasure of eating well, such as meal preparation, could reduce eating difficulties. A group of parents (n = 305) of children aged between 6 and 12 years (53.8% boys) answered a questionnaire about coercion, restriction, pleasure with eating, feeding difficulty and pleasure in preparing food. A negative correlation was found between feeding difficulty and pleasure with eating and food preparation, and a positive correlation with coercion and restriction.

#### Presence of an underlying organic disease

When evaluating children and adolescents with feeding difficulties, the presence of an underlying organic disease should always be considered, as this requires a specific diagnostic and therapeutic approach. The initial approach should include taking a targeted medical history and conducting a physical examination to look for indicators of an organic cause, such as growth failure, weight loss, persistent vomiting, dysphagia, frequent choking, pain during feeding, chronic diarrhea, severe constipation, delayed neuropsychomotor development, recurrent respiratory infections and a history of prematurity or prolonged hospitalizations [2]. These findings should direct further investigation towards gastrointestinal disorders (e.g. gastro-esophageal reflux disease, food allergies, coeliac disease), neurological disorders (e.g. cerebral palsy, swallowing disorders), metabolic disorders and cardiopulmonary disorders [2,26].

When there is clinical suspicion of an underlying organic disease, basic laboratory tests (e.g. a complete blood count, iron levels and inflammatory markers), specific tests (e.g. serology for coeliac disease) and additional tests appropriate to the clinical presentation (e.g. gastrointestinal endoscopy or pH monitoring) should be performed [2,26]. In cases where neurological or oral motor impairment is suspected, a speech and language therapy evaluation is essential to investigate dysphagia and alterations in suction-swallowing coordination [2]. The indiscriminate ordering of tests should be avoided.

Once an underlying condition is confirmed, treatment should target its cause. For example, treatment may involve controlling gastroesophageal reflux, eliminating allergens in cases of food allergy, following a gluten-free diet in cases of celiac disease, or managing neurological and metabolic conditions [2,26]. For chronic or complex diseases, treatment plans should be developed in collaboration with specialists, such as pediatric gastroenterologists, neurologists, and nutritionists.

Adequate nutritional support must also be ensured, including growth monitoring, adjustment of caloric density and/or micronutrient intake, and supplementation (medicinal or dietary) or enteral nutrition when necessary [2]. The approach should be individualized, considering the severity of the disease, nutritional impact, and capacity for oral intake.

Even after the disease is under control, feeding difficulties may persist, necessitating behavioral guidance and, eventually, a multidisciplinary approach [2,51].

#### Typical neophobia in young children

Food neophobia, or the refusal of new or unfamiliar foods, is a developmental phenomenon in young children that peaks between ages 2 and 6. It is a transient physiological condition often associated with the development of autonomy and adaptive protective mechanisms [2,27].

A clinical diagnosis is made based on selective food refusal without significant nutritional repercussions and adequate growth and development. The medical history should confirm sufficient caloric intake and the absence of warning signs, such as weight loss, dysphagia, or persistent vomiting. The dietary pattern should be restricted primarily to familiar foods [2].

Clinical management is conservative and centered on family counseling. The main therapeutic strategy consists of repeated, non-coercive exposure to foods; 8 to 15 offers are often required for acceptance [2].

Responsive feeding should be encouraged by respecting signs of hunger and satiety. Negative practices such as pressure, blackmail, or immediate substitutions with preferred foods should be avoided [2,52].

Family involvement is essential. Parents and caregivers should act as role models by sharing meals and offering a variety of foods in a structured, distraction-free environment. New foods should be introduced gradually, paired with foods that have already been accepted, and presented in an age-appropriate manner [27].

In the absence of warning signs, there is no indication for additional tests or pharmacological interventions. Follow-up should focus on monitoring growth and changes in eating patterns [2].

Thus, typical food neophobia should be recognized as part of normal development. This approach avoids unnecessary medicalization and prioritizes simple, effective educational interventions.

#### Micronutrient deficiency (hidden hunger)

Hidden hunger is characterized by an apparently adequate caloric intake yet a deficiency in essential micronutrients, such as iron, zinc, vitamins A and D, and B vitamins. This condition is common in children and adolescents with eating difficulties who have restrictive, monotonous, or inadequate diets [2,53].

A clinical-nutritional diagnosis is made by identifying a diet of low nutritional density, which may or may not be accompanied by nonspecific signs such as fatigue, pallor, increased susceptibility to infections, skin changes, or poor school performance. Since anthropometric measurements may be normal, a qualitative dietary assessment is important [2].

In addition to their systemic consequences, some micronutrient deficiencies can directly cause or contribute to feeding difficulties. For example, iron deficiency is associated with anorexia, irritability, and reduced interest in food. Zinc deficiency can lead to reduced appetite and changes in taste perception, making it difficult for children to accept food. Low levels of vitamin A can compromise mucosal integrity and taste perception. Vitamin D deficiency has also been associated with fatigue and reduced willingness to eat. Deficiencies in B vitamins, especially B12 and folate, can present with neurological changes and reduced appetite [53].

The evaluation should include a detailed dietary history, focusing on the diversity and quality of foods consumed. Laboratory tests should be ordered selectively, particularly to investigate iron deficiency anemia (complete blood count and ferritin levels), vitamin D levels, and zinc and vitamin B12 levels when clinically indicated [53].

General clinical management is based on dietary adjustments to increase nutritional density by including fresh or minimally processed foods such as fruits, vegetables, legumes, meats, eggs, and dairy products. Strategies include food fortification and meal planning [2].

Micronutrient supplementation should be initiated when a deficiency is confirmed or strongly suspected, and it may contribute to improved appetite and eating behavior. Family nutrition education is essential for promoting sustainable changes [53].

Follow-up should include clinical monitoring, laboratory testing when indicated, and evaluation of the therapeutic response. Recognizing and treating hidden hunger is fundamental to preventing nutritional deficits and breaking the cycle that perpetuates eating difficulties [2].

#### Difficulty concentrating on food

Studies on eating behavior in early childhood indicate that this presentation is often associated with a pattern of high responsiveness to the environment, in which the child prioritizes external stimuli (play, social interaction, and exploration) to the detriment of feeding. This phenotype is particularly common among older infants and preschoolers, a period in which there is an intensification of autonomy and curiosity, concomitant with a physiological slowdown in growth, which already reduces energy needs and appetite [45,54].

These children typically have a high level of activity, marked curiosity, and less interest in food. When they show hunger, they tend to be satiated quickly, consuming small amounts of food and abandoning the meal early, which often makes it difficult to stay at the table. From a behavioral point of view, this pattern may be related to relatively preserved appetite self-regulation mechanisms, but also to environmental factors, such as distractions during meals and inappropriate parental eating practices, including poor schedule structure, absence of routines, and difficulty in imposing limits [27,55].

It is important to emphasize that children with this profile can be mistakenly interpreted as having neurodevelopmental disorders, especially attention deficit hyperactivity disorder (ADHD), due to high motor activity and difficulty in remaining seated during meals. However, clinical differentiation is fundamental, since, in most cases, it is a variation in eating behavior within the spectrum of normality. Still, careful evaluation should exclude associated conditions such as ASD, motor disorders, or rare neurological diseases (e.g., X-linked adrenoleukodystrophy), especially when there are other signs of developmental delay or regression of skills [2].

In addition, evidence suggests that the family environment plays a central role. The absence of responsive parenting practices — characterized by a balance between structured food supply and respect for the child's internal hunger and satiety signals — can contribute to dysfunctional eating patterns. On the other hand, interventions based on parental education, establishment of consistent eating routines, and reduction of distractions during meals demonstrate positive results in improving food intake and behavior at the table [56,57].

#### Food phobia

Food neophobia is defined as a fear of new foods and is recognized as a normal part of development, typically peaking between the ages of 2 and 6, as children develop independence and food preferences [52,58]. The distinction between typical neophobia and selective eating depends on severity, persistence beyond the expected developmental window, and functional impact on nutrition and psychosocial functioning.

#### Hyporexia

a) Slow growth phase

There is often the idea that infant growth follows a continuous and smooth curved line, but the process is much more dynamic. The most emblematic study on the subject was published by Lampl et al., showing that growth occurs in short and intense episodes, which last about 24 h [59]. Between these jumps, intervals of 2 to 63 days can occur in which there is no measurable growth [59]. This is due to the fact that growth is mediated by hormones (such as GH - growth hormone) that are not released constantly, but rather in peaks, usually associated with specific biological processes [60]. For this reason, during periods of growth stagnation, due to the lower energy requirement, it is possible that there will be a physiological reduction in hunger [45].

b) Use of drugs that reduce hunger

The reduction of hunger and appetite in children and adolescents associated with the use of certain drugs is mainly due to the pharmacological modulation of the central and peripheral systems that regulate energy homeostasis, especially at the level of the hypothalamus and reward circuits. Psychostimulant drugs widely used in attention-deficit/hyperactivity disorder (ADHD), such as methylphenidate and lisdexamfetamine, increase the synaptic availability of dopamine and noradrenaline, promoting anorectic effect by activating catecholaminergic pathways and suppressing orexigenic signals, being associated with a significant increase in the risk of decreased appetite and weight loss in pediatric populations [[61], [62], [63]]. Antidepressants such as selective serotonin reuptake inhibitors (SSRIs), including fluoxetine and sertraline, may reduce appetite by increasing serotonergic neurotransmission, which activates hypothalamic anorectic pathways (particularly via 5-HT2C receptors), with decreased appetite being a frequently described adverse effect in children and adolescents using these medications [64,65]. In addition, drugs used in the treatment of obesity or metabolic disorders, such as GLP-1 receptor agonists, topiramate and metformin, act directly on central satiety pathways, contributing to a reduction in food intake [66].

c) Presence of a psychic condition

Underlying psychic conditions can lead to loss of hunger (hyporexia) in children and adolescents through neurobiological and behavioral mechanisms that involve dysregulation of the hypothalamic–pituitary–adrenal axis, changes in the signaling of neurotransmitters such as serotonin and dopamine, and modifications in food reward circuits. These processes often involve interaction between psychological, neurobiological, and environmental factors, resulting in sustained reduction in dietary intake and nutritional risk. Depressive disorders, for example, are associated with reduced appetite due to anhedonia and decreased responsiveness to food reinforcement, while anxiety disorders can co-occur with chronic activation of the stress system, increasing cortisol levels and reducing food intake. Eating disorders, such as anorexia nervosa, have specific cognitive-behavioral mechanisms (intense fear of weight gain, distortion of body image) associated with neuroendocrine adaptations that suppress hunger. In addition, autism spectrum disorder and attention-deficit/hyperactivity disorder (ADHD) may co-occur with hyperoxia related to behavioral rigidity, food selectivity, or difficulty in interoceptive self-regulation. Conditions such as obsessive-compulsive disorder and chronic psychosocial stress can also negatively interfere with food intake through increased anxiety and avoidant behaviors [7,[67], [68], [69], [70], [71]].

In these cases, the treatment of the underlying disease is fundamental, whether through psychotherapy, the use of drugs or both. Often, the therapeutic response can be slow and late and, in these cases, the use of complete supplementation should be considered, while waiting for the improvement of the psychic condition [72].

#### Food selectivity

Food selectivity represents one of the most prevalent and challenging presentations within the spectrum of feeding difficulties in childhood, characterized by a restricted dietary repertoire based on food type, texture, presentation, or brand [2]. Unlike the transient neophobia commonly observed in typically developing toddlers, pathological selectivity persists beyond expected developmental windows, significantly impacts nutritional adequacy, and generates substantial family distress [27,73]. The heterogeneity of selective eating—ranging from mild preferences to severe food refusal requiring enteral nutrition—demands a structured, treatment-oriented approach that guides clinicians through progressive levels of intervention complexity [74].

a) Evaluation of Selectivity Severity

For children with and without ASD, the algorithm requires assessment of selectivity severity based on multiple parameters: number of accepted foods (with severe selectivity typically defined as fewer than 20 foods or exclusion of entire food groups), presence of anthropometric compromise (weight-for-height or BMI below the 5th percentile, or crossing two major percentile lines), laboratory evidence of nutritional deficiency, and degree of functional impairment (inability to participate in social eating, family distress, school difficulties) [75,76].

Mild to moderate selectivity — characterized by a restricted but nutritionally adequate diet, normal growth, and manageable family impact — can often be addressed through outpatient nutritional counseling and behavioral guidance provided by dietitians and psychologists with expertise in pediatric feeding [77]. Interventions at this level include systematic desensitization protocols, parent-mediated exposure strategies, and nutritional optimization of the accepted food repertoire through fortification and supplementation [78,79].

Severe selectivity, particularly when accompanied by growth faltering, micronutrient deficiencies, or extreme dietary restriction (fewer than 10–15 foods), necessitates referral to specialized ARFID programs or multidisciplinary feeding clinics [80,81]. These programs integrate medical management (pediatric gastroenterology or nutrition medicine), nutritional rehabilitation (specialized pediatric dietitians), behavioral intervention (psychologists trained in feeding disorders), oral-motor therapy (speech-language pathologists and occupational therapists), and family support [82,83].

b) Differentiation of Sensory Subtypes: Hyposensorial versus Hypersensorial Presentations

One of the major strengths of the FEEDSETUP framework is the explicit distinction between hypersensorial (sensory over-responsive) and hyposensorial (sensory under-responsive) feeding phenotypes. Although both may present clinically as food selectivity, the underlying neurophysiological mechanisms, observable behaviors, and therapeutic needs differ substantially. Failure to distinguish these sensory profiles may lead to interventions that are ineffective or even counterproductive [84,85]. Current models of sensory processing propose that feeding behavior results from the interaction between neurological thresholds for sensory input and self-regulation strategies. Children with low neurological thresholds detect sensory stimuli rapidly and intensely, resulting in sensory avoidance behaviors, whereas children with high neurological thresholds require stronger sensory stimulation before responding and often demonstrate sensory-seeking behaviors. These differences are particularly common among children with ASD but may also occur in children with isolated sensory processing disorder, developmental delay, ADHD, and some typically developing children [86].

### Hypersensorial (sensory over-responsive)

Children with hypersensory processing exhibit exaggerated neural responses to ordinary sensory stimuli. During meals, textures, smells, temperatures, colors or even the appearance of foods may be perceived as unpleasant or overwhelming [87]. Rather than simple food preferences, these children frequently experience genuine sensory discomfort. They may demonstrate strong aversive reactions (gagging, vomiting, behavioral distress) to specific textures (commonly mixed textures, slimy or sticky foods) or strong flavors [88]. The accepted food repertoire often consists of bland, predictable foods with uniform texture (crackers, plain pasta, specific brands of chicken nuggets) [89].

### Typical clinical examples

A hypersensorial child may: (1) eat only crispy foods such as crackers, toast or potato chips; (2) refuse pasta if the sauce touches it; (3) accept chicken nuggets from only one manufacturer because slight differences in coating or texture are detected; (4) gag immediately when mashed potatoes or bananas enter the mouth; (5) refuse foods solely because of their smell before tasting them; and (6) become distressed when different foods touch each other on the plate. The feeding pattern is therefore characterized more by avoidance of aversive sensory input than by lack of appetite.

### Therapeutic implications for hypersensorial feeding

Treatment focuses primarily on reducing sensory defensiveness while gradually increasing tolerance, rather than immediately increasing food intake. One of the most supported approaches involves graded sensory exposure, in which the child progresses through a hierarchy of increasingly demanding interactions with food [90,91]. Occupational therapy, particularly when integrated with sensory-based strategies, graduated food exposure, caregiver coaching, and behavioral interventions, forms an important component of multidisciplinary treatment. Current evidence suggests that multimodal interventions tailored to the child's sensory profile are more consistently effective than isolated sensory integration techniques alone. Food Chaining and systematic desensitization may facilitate acceptance of novel foods, although the strength of evidence varies across intervention models [92,93]. Nutritional management focuses on identifying foods within the child’s sensory tolerance that maximize nutritional density, along with targeted supplementation to address micronutrient gaps [94].

Environmental modifications are critical: reducing sensory overload at mealtimes (controlling noise, visual stimulation, and olfactory input), using preferred utensils and dishes, and maintaining predictable mealtime routines [95]. Parent education emphasizes the neurobiological basis of sensory sensitivity to reduce pressure and anxiety, which can exacerbate sensory defensiveness [96]. Therefore, the progression may include: (1) tolerating the food in the room; (2) looking at the food; (3) touching it with utensils; (4) touching with fingers; (5) smelling; (6) kissing or licking; (7) placing the food in the mouth; (8) chewing; and (9) swallowing.

Repeated, low-pressure exposure allows habituation of sensory responses and reduces anticipatory anxiety. Importantly, advancement depends on the child's tolerance rather than forced consumption.

### Hyposensorial (or sensory under-responsive)

In contrast, children with hyposensory processing exhibit diminished responsiveness to ordinary sensory input. Because oral sensations are insufficiently salient, they often seek stronger sensory experiences during eating [97]. In the feeding context, these children may prefer foods with strong flavors (very salty, spicy, or sour), crunchy or chewy textures that provide proprioceptive feedback, or extreme temperatures [98]. They may appear disinterested in eating, require external cues to initiate and maintain eating, and demonstrate poor awareness of hunger and satiety signals [99].

### Typical clinical examples

A hyposensorial child may: (1) strongly prefer pretzels, carrots, beef jerky or toasted bread because of their crunchiness; (2) constantly chew shirt collars, pencils or silicone chew toys; (3) request very spicy, salty or sour foods compared with peers; (4) keep food in the mouth for prolonged periods ("packing"); (5) fail to notice food remaining on the lips or face; (6) forget to eat unless reminded; and (7) appear unaware of hunger until becoming irritable. Unlike hypersensory children, these children generally tolerate a broad variety of foods but seek foods that generate greater sensory input.

### Therapeutic implications for hyposensorial feeding

Treatment for hyposensorial selectivity focuses on increasing sensory input and awareness through foods that provide enhanced sensory feedback [100]. Sensory strategies frequently include crunchy foods, chewy textures, temperature contrasts, stronger flavors, foods requiring greater oral work and oral sensory activities immediately before meals when clinically indicated [101]. Occupational therapy interventions may include oral-motor exercises, use of vibrating or textured utensils, and activities to increase overall sensory awareness and body awareness [102]. Behavioral strategies emphasize external structure and cueing, as these children may not respond to internal hunger signals [103]. Scheduled meals and snacks, visual supports (timers, portion indicators), and active engagement during meals (minimizing distractions, encouraging self-feeding with sensory-rich foods) are important components [104].

### Distinguishing the two phenotypes in clinical practice

Although hypersensorial and hyposensorial presentations represent opposite ends of sensory responsiveness, they should not be viewed as mutually exclusive categories. Many children demonstrate mixed sensory profiles, exhibiting hypersensitivity in one sensory modality (for example, texture) while simultaneously seeking sensory input through another modality (such as strong flavors or excessive chewing). Consequently, comprehensive assessment by an interdisciplinary team is essential before treatment planning. Table 3 illustrates the differences in clinical characteristics between the two forms of selectivity.

**Table 3:** The hypersensorial (sensory over-responsive) and hyposensorial (sensory under-responsive) phenotypes based on Clinical Practice.

Characteristic	Hypersensorial	Hyposensorial
Neurological threshold	Low	High
Primary behavior	Sensory avoidance	Sensory seeking
Typical food preference	Bland, predictable, uniform	Crunchy, chewy, intense flavors
Common mealtime behavior	Gagging, refusal, distress	Slow eating, low awareness, excessive chewing
Main therapeutic goal	Reduce sensory defensiveness	Increase sensory input and awareness
Exposure strategy	Gradual desensitization	Progressive sensory enhancement
Behavioral focus	Reduce escape and anxiety	Increase engagement and meal initiation
Occupational therapy	Sensory modulation and tolerance	Sensory activation and interoceptive awareness

### Integrating therapy within the feedsetup model

Within the FEEDSETUP framework, identifying whether food selectivity primarily reflects hypersensory or hyposensory processing guides individualized intervention planning. Sensory-based approaches alone are rarely sufficient. Optimal management combines occupational therapy, feeding therapy, behavioral interventions, caregiver coaching and nutritional management. Current evidence increasingly supports individualized, multidisciplinary interventions that integrate sensory-based approaches with behavioral principles rather than relying on either approach alone. Selection of therapeutic strategies should be guided by comprehensive assessment of the child's sensory processing profile, developmental abilities, nutritional status, and family context.

For hypersensory children, therapy prioritizes reducing sensory threat through graded exposure while maintaining nutritional adequacy. For hyposensory children, intervention emphasizes enhancing oral sensory feedback and improving self-regulation of eating behaviors. In both cases, caregiver education is fundamental to avoid coercive feeding practices, reduce mealtime stress and promote consistent implementation of therapeutic strategies across home, school and clinical settings.

### Treatment pathways

The FEEDSETUP framework addresses a critical gap in existing classification systems by prioritizing treatment pathways over purely diagnostic categorization [105]. While previous frameworks such as the DSM-5 ARFID criteria and the Pediatric Feeding Disorder (PFD) consensus definition have advanced the understanding of feeding difficulties, they remain primarily diagnostic constructs that do not explicitly delineate when and how to escalate care [1,106] The FEEDSETUP algorithm operationalizes a stepwise approach wherein the intensity and specialization of intervention are matched to the severity and complexity of the clinical presentation, ensuring that resources are allocated efficiently while avoiding both under-treatment of severe cases and over-medicalization of mild variants [9]

a) Primary Healthcare as the First Line of Assessment and Intervention

A fundamental principle of the FEEDSETUP framework is the central role of primary healthcare professionals — pediatricians, family physicians, and primary care nurses—as the initial evaluators and managers of food selectivity [107]. This positioning reflects both epidemiological reality and practical necessity: feeding difficulties affect 25–45% of typically developing children and up to 80% of those with developmental disorders, making specialized referrals for all cases neither feasible nor appropriate [9,11]. Primary care providers are uniquely positioned to distinguish between normal developmental variants (such as physiological neophobia in toddlers aged 18–24 months) and pathological selectivity requiring intervention [52].

The initial assessment in primary care encompasses comprehensive history-taking, including onset and duration of selectivity, accepted food repertoire (ideally quantified as number of foods across food groups), mealtime behaviors, growth trajectory, and developmental milestones [108]. Physical examination focuses on anthropometric measurements (weight, height, weight-for-height or BMI), signs of micronutrient deficiencies (pallor, glossitis, angular cheilitis, dermatological changes), and oral-motor function [109]. Laboratory screening, when indicated by clinical findings, may include complete blood count, ferritin, vitamin D, and zinc levels, as deficiencies in these micronutrients are common in selective eaters and may perpetuate feeding difficulties through effects on appetite and taste perception [110,111].

For mild to moderate selectivity without growth impairment or micronutrient deficiency, primary care management includes family education on normal eating development, guidance on responsive feeding practices (avoiding pressure, maintaining mealtime structure, repeated neutral exposure to novel foods), and nutritional counseling to optimize the accepted food repertoire [50,112]. This first-line approach is effective for a substantial proportion of cases, particularly when initiated early and when parental anxiety is addressed through reassurance and practical strategies [113].

b) Progressive Involvement of Specialized Professionals

The FEEDSETUP algorithm delineates clear criteria for escalation to specialized care, ensuring that children with more complex or severe presentations receive timely multidisciplinary evaluation [114]. The selectivity pathway incorporates two critical decision points that determine the level of intervention required: (1) the presence or absence of ASD or other neurodevelopmental conditions, and (2) the severity of selectivity and its functional consequences [115].

c) Assessment of Autism Spectrum Disorder

The evaluation of ASD comorbidity is a pivotal branch point in the selectivity pathway, as feeding difficulties occur in 46–89% of children with ASD, with selectivity being the most common presentation [116,117]. Children with ASD demonstrate significantly greater food refusal, limited food repertoire (often fewer than 20 accepted foods), and rigid food-related rituals compared to neurotypical peers [118]. The underlying mechanisms are multifactorial, including sensory processing differences, insistence on sameness, anxiety, and oral-motor difficulties [119,120].

When ASD is present or suspected based on developmental screening, referral to developmental-behavioral pediatrics or child psychiatry for diagnostic evaluation is indicated [121]. Concurrent assessment by occupational therapy for sensory processing evaluation and speech-language pathology for oral-motor function is often warranted [122]. The presence of ASD fundamentally alters the treatment approach, as interventions must account for the child’s sensory profile, need for predictability, and communication style [123].

d) Referral to ARFID Specialists and Multidisciplinary Teams

The FEEDSETUP algorithm specifies clear criteria for referral to specialized ARFID programs and multidisciplinary feeding teams, ensuring that children with the most severe and complex presentations receive intensive, coordinated care [124] Indications for specialized referral include:

**1) Severe dietary restriction**: Fewer than 10–20 foods accepted, or complete exclusion of multiple food groups, with inadequate nutritional intake despite outpatient intervention [125].
**2) Growth faltering or malnutrition**: Weight-for-height or BMI below the 5th percentile, loss of more than two major percentile lines, or laboratory evidence of protein-energy malnutrition or multiple micronutrient deficiencies [126].
**3) Medical complications**: Electrolyte abnormalities, cardiac complications (bradycardia, orthostatic hypotension), or other medical sequelae of malnutrition requiring medical stabilization [127].
**4) Comorbid medical or psychiatric conditions**: Presence of gastrointestinal disorders (eosinophilic esophagitis, inflammatory bowel disease), neurodevelopmental disorders (ASD, intellectual disability), or psychiatric conditions (anxiety disorders, obsessive-compulsive disorder) that complicate feeding management [128,129].
**5) Failed outpatient treatment**: Lack of progress or deterioration despite appropriate outpatient intervention over 3–6 months [130].
**6) Extreme behavioral rigidity**: Food-related rituals, brand-specific requirements, or anxiety so severe that it prevents exposure-based intervention in less intensive settings [131].

Multidisciplinary feeding teams typically include pediatric gastroenterologists or nutrition medicine specialists (medical management, exclusion of organic pathology), pediatric dietitians (nutritional assessment and rehabilitation), psychologists or behavioral specialists (behavioral intervention, anxiety management, parent training), speech-language pathologists (oral-motor assessment and therapy), occupational therapists (sensory processing evaluation and intervention), and social workers (family support, care coordination) [132,133].

Treatment modalities in specialized programs may include intensive outpatient therapy (multiple sessions per week), partial hospitalization programs (day treatment), or inpatient admission for medical stabilization and intensive behavioral intervention [134]. Evidence-based behavioral interventions include escape extinction protocols, differential reinforcement, systematic desensitization, and parent-mediated interventions adapted to the child’s developmental level and sensory profile [135,136]. Table 4 shows the criteria for escalation from primary to specialized care. Table 5 shows the multidisciplinary team roles and timing of involvement. Care is organized around a single shared plan, with the pediatrician as the constant point of reference; each specialist returns a written assessment and explicit criteria for de-escalation to primary care follow-up.

e) Integration and Continuous Care

**Table 4:** Criteria for scalation from primary to specialized care.

Criterion	Operational threshold	Level of care
Growth	Weight-for-height or BMI-for-age < P5, or crossing of two major percentile channels	Specialized team
Weight change	Weight loss, or no weight gain over 3 consecutive months	Specialized team
Repertoire	< 10–20 accepted foods, or exclusion of an entire food group	Specialized team
Nutritional status	Confirmed micronutrient deficiency or signs of protein-energy malnutrition	Pediatrician + dietitian; team if severe
Feeding route	Enteral dependence or inability to sustain oral intake	Specialized team, urgent
Medical stability	Electrolyte disturbance, bradycardia, orthostatic hypotension, dehydration	Hospital assessment, urgent
Distress and function	Exclusion from family or school meals; meals routinely > 40 min or conflictual; significant family distress	Pediatrician + psychologist
Treatment response	No progress after 3–6 months of appropriate first-line management	Specialized team
Comorbidity	Suspected or confirmed ASD, developmental disability or ARFID	Specialized team, with continued pediatric follow-up

**Table 5:** Multidisciplinary team: roles and timing of involvement.

Professional	Role in the FEEDSETUP pathway	When to involve
Pediatrician / family physician	History, anthropometry, initial workup, classification, coordination and follow-up	Always; entry and constant point of reference
Pediatric gastroenterologist	Organic causes: reflux, allergy, dysphagia, structural and motility disorders	Suspected organic disease, failure to thrive, pain or vomiting
Pediatric dietitian	Dietary assessment, energy and micronutrient adequacy, supplementation, repertoire expansion	Any nutritional inadequacy or restricted repertoire
Psychologist	Anxiety, aversive conditioning, family dynamics; behavioral intervention and parental guidance	Food phobia, mealtime conflict, suspected ARFID
Occupational therapist	Sensory profile, sensory-based intervention, mealtime adaptation	Hyper- or hyposensorial presentations
Speech-language pathologist	Oral-motor and swallowing assessment, texture progression, intake safety	Suspected dysphagia, oral-motor delay, choking or coughing
Feeding therapist	Structured intervention, graded exposure, food chaining	Severe selectivity; intensive programmes
Social worker	Food insecurity, access to services, family support	Suspected food insecurity or social vulnerability

A critical strength of the FEEDSETUP framework is its emphasis on bidirectional communication and care coordination across levels of intervention [137]. Primary care providers remain central throughout the treatment trajectory, receiving consultation from specialists, monitoring growth and development, and managing the transition back to primary care as the child stabilizes [138]. This integrated model ensures continuity, prevents fragmentation of care, and supports families in navigating complex healthcare systems [139].

The selectivity pathway within the FEEDSETUP algorithm thus operationalizes a comprehensive, evidence-based approach that matches intervention intensity to clinical need, leverages the expertise of primary care providers while ensuring timely access to specialized care, and addresses the multifactorial nature of food selectivity through coordinated multidisciplinary intervention [14] By explicitly incorporating assessment of neurodevelopmental comorbidities, severity stratification, and sensory subtyping, the framework provides clinicians with a practical roadmap for managing one of the most common and challenging presentations in pediatric feeding difficulties.

f) FEEDSETUP across diverse healthcare systems and cultural contexts

FEEDSETUP was developed based on evidence from various clinical settings. However, it should be adapted to each region's healthcare system, cultural context, and available resources. Feeding behaviors are strongly influenced by cultural food practices, family beliefs, socioeconomic conditions, food availability, and caregiver expectations. These factors shape children's eating experiences and perceptions of feeding difficulties. Thus, assessments of food selectivity, neophobia, and other feeding problems should be interpreted within the context of culturally normative dietary practices rather than against a universal standard. Similarly, intervention strategies should be tailored to incorporate locally available foods, culturally accepted eating patterns, and family routines, all while preserving the framework's core principles. FEEDSETUP's stepwise structure facilitates its application across different healthcare settings, allowing management to be tailored to local resources. This ranges from primary care services with limited specialist access to multidisciplinary tertiary centers. This flexibility enhances the framework's external validity and supports its use in diverse populations while maintaining a standardized approach to assessing and managing pediatric feeding difficulties.

## Conclusions

The FEEDSETUP framework represents a significant advancement in the understanding and management of pediatric feeding difficulties, moving beyond fragmented diagnostic labels towards a comprehensive, spectrum-based approach. By integrating a systematic initial assessment with a treatment-focused classification, FEEDSETUP provides a clear, evidence-based roadmap for clinicians. This model ensures that intervention intensity is appropriately matched to the severity and complexity of each case, from primary care management of mild difficulties to specialized multidisciplinary interventions for severe presentations, including those complicated by neurodevelopmental comorbidities like ASD or severe ARFID. The emphasis on coordinated care, early identification, and tailored interventions is crucial for improving outcomes, reducing diagnostic delays, and alleviating the significant family distress often associated with these challenges. Ultimately, FEEDSETUP operationalizes a practical and integrated strategy that addresses the multifactorial nature of feeding difficulties, fostering more effective and individualized care for children and adolescents across the entire spectrum of eating challenges.

### Limitations, future directions and implementation considerations

FEEDSETUP is a proposal derived from narrative synthesis and expert consensus and has not yet been prospectively validated. Its evidence base is uneven across domains, and its execution presupposes multidisciplinary teams which are unavailable in much of the public health network. It requires training of primary care professionals in recognizing sensory phenotypes. In resource-limited settings, the escalation pathways may be aspirational rather than practicable. Prospective validation of the algorithm in primary care cohorts is desirable, along with assessment of inter-rater reliability of the classification among pediatricians, dietitians and therapists. In public health services, feasibility and acceptability studies need to be conducted, and outcome studies will be important for examining whether use of the framework improves nutritional status, dietary repertoire, quality of life for child and family, time to appropriate referral, and healthcare utilization.

## Declaration of generative AI and AI-assisted technologies in the manuscript preparation process

During the preparation of this work, the author(s) used Gemini (Google Inc.) to help with the creation of Figure 1. After using this tool/service, the author(s) reviewed and edited the content as needed and take(s) full responsibility for the content of the published article.

## Data availability

The data that support the findings of this study are available from the corresponding author.

## Funding

No funding is disclosed for this paper.

## Conflicts of interest

The authors declare that the research was conducted in the absence of any commercial or financial relationships that could be construed as a potential conflict of interest.
